# Correlation of Pulp Calcification and Cardiovascular Conditions: A Literature Review

**DOI:** 10.7759/cureus.47258

**Published:** 2023-10-18

**Authors:** Parul R Loya, Pradnya P Nikhade

**Affiliations:** 1 Conservative Dentistry and Endodontics, Sharad Pawar Dental College and Hospital, Datta Meghe Institute of Higher Education and Research (DMIHER), Wardha, IND

**Keywords:** systemic diseases, radiography, pulp stones, cardiovascular disease, pulp calcification

## Abstract

The pathophysiology of calcified dental pulp is considered to be comparable to that of calcified atheroma in the artery. These calcified masses are small nodular which is seen more often in the coronal pulp region than in the radicular pulp. This is generally more common in the elderly population and usually corresponds to increasing age. Calcifications are also found in the brain, breast, arteries, and kidneys. There is a link between pulp calcification and cardiovascular problems. It is commonly assumed that individuals suffering from cardiovascular diseases have a higher risk of calcification in the pulp. The use of radiography as a quick means of detecting cardiovascular disease is possible. The pulp calcification process is usually triggered by the osteoblastic process. The process is identified by the presence of an osteoid matrix, which is built down by odontoblast cells in the pulp’s peripheral portions, culminating in the production of tissue that is comparable to dentine. This review will look at pulp calcifications from all angles, including their mechanism, clinical considerations, radiographic features, and management, and also to determine if there is a link between pulp calcification and cardiovascular problems.

## Introduction and background

The collection of calcium salts in different parts of the body is known as calcification. It is usually collected in soft tissue which slowly becomes solid. The dental pulp is a soft tissue that is surrounded by a tooth’s hard structures. Pulp stones or denticles are calcification as whole or discrete masses which the pulp itself experiences. Pulp stones are nodular, calcified masses seen more commonly in coronal pulp than in radicular pulp [1]. Pulp stones can differ from small structures embedded in pulp tissue to large masses. They are more frequently noticed in molars. They can be free, attached, and embedded.

Hamasha and Darwazeh concluded the observation that molars are the largest teeth in the arch, therefore there will be an increase in blood supply which would result in increased precipitation of calcium in posterior teeth [2]. Pulp stones occur due to pulp degradation, aging, caries, periodontal diseases, orthodontic tooth movement, genetic predisposition, and trauma [1,3]. There are occurrences in which the whole pulp chamber gets calcified with pulp stones and the canals get completely narrowed. This situation is mainly seen in elderly people and can be correlated with increasing age. It is generally seen that individuals suffering from cardiovascular diseases have a high risk of pulp stones [4]. Calcification can also be seen in joints, arteries, kidneys, breasts, pericardium, tendons, and brain. Cardiovascular disease tends to damage the innermost layer of the blood vessels which is the endothelium due to the formation of plaque within it.

Any individual with cardiovascular disease who is suffering from myocardial infarction, peripheral artery disease, angina pectoris coronary heart disease, usually results from the thickening or stiffening of heart muscles or atherosclerosis. Cardiovascular disease tends to damage the innermost layer of the blood vessels which is the endothelium due to the formation of plaque within it. A new constitute of atherosclerotic plaque, known as osteopontin (OPN) plays a crucial role in plaque formation [5,6]. Due to the accumulation of calcified plaque, the arteries become thick and get stiff which decreases the blood circulation to essential organs like the brain and heart which further causes a stroke. In India’s population, cardiovascular disorders are the most common, followed by diabetes mellitus. Only a few studies have looked at the extent and character of healthcare availability among Nepali migrant workers in India [7]. Obesity has reached the top of the list of the most dangerous non-communicable diseases. It has a direct link to cardiovascular health in people of all ages [8].

According to Maranhao de Moura and de Paiva, coronary artery disease patients have a higher pulp calcification rate [9]. According to the literature, Edds et al. [10] concluded 74%, and Khojastepour et al. [11]. recorded 68.2% of pulp stones present in the teeth of cardiovascular disease patients. Khojastepour et al. also recorded that 28.2% of pulp stones are present in the teeth of non-cardiovascular disease patients [11]. The absolute and age-standardized estimates show patterns that are similar to those seen in high blood pressure, which is linked to hypertension and heart disease [12].

 Early recognition of vascular calcification with the use of different imaging techniques like radiography and ultrasound helps to reach certain conclusions and early intervention is possible. There is a definite correlation between pulpal calcification and cardiovascular episodes which can be assessed using radiographic techniques to identify the pulpal calcification and co-relate it with future cardiovascular diseases [13-15]. A dental x-ray known as an Orthopantomogram (OPG) creates images of both the upper arch and lower arch. Radiographic findings include calcifications like the occurrence of pulp stones and the complete narrowing of root canals of teeth. In radiographs, pulp stones appear as ovoid or round opacities as well as completely opaque canals of the teeth [16,17]. Thus, oral and maxillofacial radiology might be helpful in the early diagnosis and co-relation of pulpal calcifications with future chances of cardio-vascular episodes. Cardiovascular disease can be detected quickly using radiography.

## Review

Methodology

An electronic comprehensive search was carried out till June 2022 for the studies using the following keywords such as pulp calcification, OPG, pulp stones, and cardiovascular disease with the following databases: EBSCOhost, Google Scholar, and PubMed to recover articles in the English language. The searches in the grey literature, cross-referencing, and clinical trials database were conducted using OpenGrey, Greylist, and Google Scholar. A manual look at different journals such as the Australian Dental Journal, American Journal of Medical Genetics, Journal of Endodontics, BMC Health Services Research, Journal of Oral Medicine Oral Pathology Oral Radiology, Journal of Endodontics, Journal of Clinical and Diagnostic Research, Oral Surgery, Iranian endodontic journal was performed. The inclusion criteria included relevant books, articles, and reviews. The study selection procedure included screening titles and abstracts, followed by a full-text evaluation of relevant papers. The final group of included research offers a thorough analysis of the evidence that is currently available on pulp calcification and its correlation with cardiovascular patients. The results were combined and analyzed to draw meaningful conclusions.

Mechanism of pulp calcification

**Figure 1:**
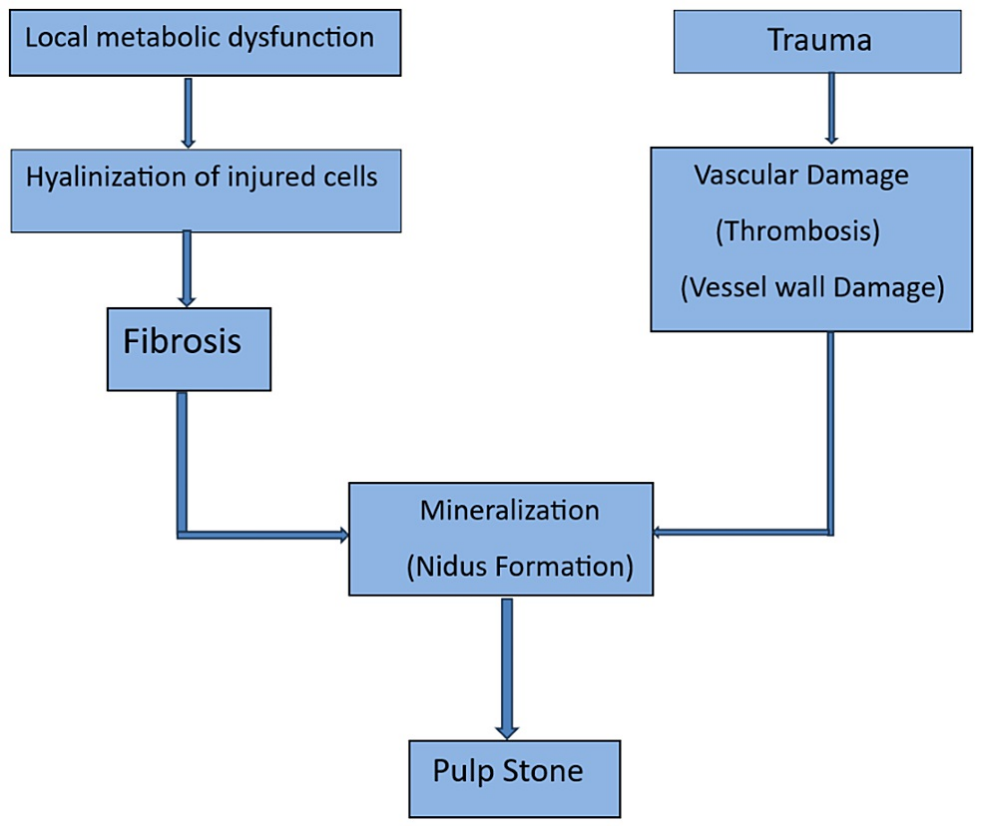
Mechanism of pulp calcification

The osteoblastic process generally triggers pulp calcification activity. The process is denoted by the occurrence of the osteoid matrix which is laid down by odontoblast cells in the peripheral areas of the pulp resulting in a concomitant subsequently, this will act as a triggering factor for the initiation of calcification in the pulp thereby causing pulpal obliteration (Figure 1).

Clinical presentation of pulp calcification

In clinical practice, patients come to the doctor with a yellowing of the affected dental crown. This color change is due to the intensity of the dentine insertion [18,19]. You must be aware that not all teeth will change color. On the other hand, greyish discoloration is seen due to trauma which causes pulp necrosis. Pulp calcification is most common on external teeth and can be seen three months after injury although it may not be available for up to one year. The incidence of tooth decay following tooth decay was reported to be estimated at 4%-24%. Unless they impinge on nerves and blood arteries, calcifications in the pulp are usually asymptomatic. Vital pulp tests may be unreliable (false) even though there is significant damage due to increased dental stiffness. Most frequently, teeth with calcified pulp appear dark yellow which may be because of tertiary dentine depositions. They can change the internal structure of the pulp cavity, making the approach more difficult. Endodontic instruments’ tips may be deflected or engaged by them. 

Radiographic presentation of pulp calcification

Pulp calcification can be seen as radiopaque structures in the entire pulp chamber or part of it, in the root canals by narrowing or obliterating, or can continue from the pulp chamber to root canals. Within the pulp chamber, trauma produces a fast deposition of hard tissue. The causes of obliteration are trauma, dental treatments, systemic factors, any associated syndromes, and drugs. It is frequently discovered by chance and patients are usually unaffected [20]. A radiograph frequently indicates pulp space obliteration or complete obliteration [21].

Partial Obliteration

The root canal will become thin but visible, and the pulp chamber will be visible. Complete obliteration-The pulp chamber and canal become mineralized to the point where they are no longer visible. The lamina dura remains intact with no expansion of the periodontal ligament area unless there is evidence of periapical bone involvement. 

Histopathology of pulp stones is generally divided into three types

Free pulp stones are seen within the pulp tissue and are commonly found. The size may vary from 50μm in diameter to several millimeters and may obstruct the entire pulp chamber. Attached pulp stones are attached to the wall of pulp space but not fully enclosed by dentine. Embedded pulp stones are fully embedded in the dentine and are most commonly found in the apical portion of the root.

Correlation of calcifications in blood vessels and cardiovascular disease

Atherosclerosis, one of the most severe diseases has been demonstrated to show less or minimal symptoms. As a result, late detection may be life-threatening. Early detection of this disease is a prime requisite to preventing strokes or cardiac arrest. A large population is affected by atherosclerosis; therefore, a dental clinician may play a significant role in the early diagnosis of the disease resulting in timely diagnosis and early treatment intervention. Incidental finding of pulp calcified stones on intra or extra-oral x-rays may be a suggestion of atherosclerosis. If this could be prevented, then it would be a major contribution to public health.

Edds et al.'s study observed that patients suffering from cardiovascular diseases show higher occurrences of dental pulp stones or calcification. Nayak et al. stated in one of their studies that a favorable link was discovered between cardiovascular disease and pulp stones or denticles. Horsley observed that when pulp calcification was present, he was able to test for carotid calcification with a 66.4% accuracy. The current study shows a screening test that was useful to find pulp calcification in radiography which helped to evaluate atherosclerosis in cardiovascular individuals (sensitivity = 68.9%), which is in agreement with earlier mentioned studies but is in contrast to one. Increased blood flow to the molar pulp tissues may result in calcium precipitation in the pulp chamber. Earlier show-up of first molars in the mouth which constantly increased stress on teeth may explicate the increased rate of pulp calcification.

Most elderly individuals >60 years old have regularly enlarging deposits of calcium minerals in their essential arteries [22]. Vascular calcification is the deposition of minerals in the vascular system. It is also found in heart valves which eventually causes more and more accumulation of calcification in the lumen causing narrowing of it resulting in low blood supply and increased blood pressure leading to stroke. Vascular calcification is a dynamic and well-controlled process that is associated with cardiovascular disease and hypertension. According to Edds et al., 74% of people with a history of cardiovascular disease had recognizable pulp stones, but only 39% of people without a history of cardiovascular disease had. This study suggests that dental radiographs can be used to identify patients with cardiovascular disease who should be further examined and evaluated.

Management

There is always a debate regarding endodontic therapy. Some authors suggest that endodontic therapy should be immediate as pulp calcification may lead to infection while other authors suggest treatment only after the appearance of symptoms. If a patient complains of discoloration, then external bleaching can be done. In case of abnormal reparative dentin formation, an intentional root canal remedy accompanied by way of nonvital bleaching is done [23]. If root canal therapy is recommended, a thorough understanding of root canal anatomy and orifice opening is essential, which can be determined using methylene blue dye or a champagne bubble test using sodium hypochlorite. Also, the DG-16 endodontic explorer is very useful for the same. It is quite beneficial for the operator to place the rubber dam before the access opening [24]. Once the canal has been found, a rubber dam must be installed immediately, as well as treatment. The access cavity must be of regular size and form [25]. The canal should be indicated by a change in the color of the dentine inside the middle of the root. In their study, Fachin and colleagues introduced the “Modified-Tip Instrument” technique for the removal of tough materials from the root canal during retreatment procedures, particularly recommending its application in cases of highly calcified canals. This method involves taking a K-type file, specifically sizes #30 and #35, and trimming its tip by 4 mm using an orthodontic wire cutter, resulting in the creation of a sharp edge at the newly formed working end. When employed with controlled apical pressure and rotary movements, this modified file becomes an effective and powerful cutting tool [26,27]. Zehnder and colleagues discovered that “Guided Endodontics” allowed for precise root canal preparation extending to the tip of the root through the use of guiding templates. This technique is a valuable innovation in endodontics. Gabriel Krastlet, the pioneer of the guided technique in endodontics, found that guided endodontic procedures are a safe and practical method for locating root canal openings. Importantly, they help reduce the risk of accidentally perforating the root in cases of calcified or metamorphosed teeth [28,29].

## Conclusions

The correlation between cardiovascular conditions and systemic diseases may predict the incidence of cardiovascular accidents in the future. The prevalence of maturity-onset diabetes mellitus is a common finding among the elderly population having coronal vascular diseases. Pulp stone prevalence varies depending on the demographic. Correlation of evidence of calcifications observed in pulp with the future prediction of cardiac diseases. Hence the incidence of pulp calcification is used to forecast the chances in the near future times of cardiovascular diseases in otherwise healthy individuals and will be used as a tool to predict the disease and will eventually help in taking necessary steps to avoid future episodes of heart disease.
